# Progestin and AdipoQ Receptor 3 Upregulates Fibronectin and Intercellular Adhesion Molecule-1 in Glomerular Mesangial Cells *via* Activating NF-κB Signaling Pathway Under High Glucose Conditions

**DOI:** 10.3389/fendo.2018.00275

**Published:** 2018-06-07

**Authors:** Yezi Zou, Zhiquan Chen, Jie Li, Wenyan Gong, Lei Zhang, Futian Xu, Lihao Chen, Peiqing Liu, Heqing Huang

**Affiliations:** ^1^Laboratory of Pharmacology & Toxicology, School of Pharmaceutical Sciences, Sun Yat-sen University, Guangzhou, China; ^2^Department of Laboratory Medicine, Guangdong Second Provincial General Hospital, Guangzhou, China

**Keywords:** progestin and adipoQ receptor 3, diabetic nephropathy, inflammatory fibrosis, NF-κB, inhibitor of nuclear factor κB kinase β

## Abstract

**Background:**

Progestin and adipoQ receptor 3 (PAQR3), is a Golgi-anchored membrane protein containing seven transmembrane helices. It has been demonstrated that PAQR3 mediates insulin resistance, glucose and lipid metabolism, and inflammation. In addition, kidney inflammatory fibrosis is an important pathological feature of diabetic nephropathy (DN). Therefore, we aimed to investigate the role of PAQR3 in diabetic kidney fibrosis as well as inflammation in DN.

**Object:**

The effect of PAQR3 on NF-κB signaling pathway, expressions of fibronectin (FN) and intercellular adhesion molecule-1 (ICAM-1) in glomerular mesangial cells (GMCs) cultured by high glucose (HG) were examined.

**Method:**

Diabetic mouse and rat models were induced by streptozotocin (STZ). GMCs were treated with HG and transfected with PAQR3 plasmids or small-interfering RNA targeting PAQR3 or NF-κB. The protein levels of FN and ICAM-1 were examined by Western blotting, and the transcriptional activity and DNA binding activity of NF-κB were measured by dual luciferase reporter assay and electrophoretic mobility shift assay (EMSA). The interaction between PAQR3 and IKKβ (inhibitor of nuclear factor κB kinase β) was analyzed by co-immunoprecipitation.

**Results:**

PAQR3 was increased in both STZ-induced diabetic models and HG-treated GMCs. PAQR3 overexpression further increased HG-induced FN and ICAM-1 upregulation. In contrast, silencing of PAQR3 suppressed the expressions of FN and ICAM-1. PAQR3 overexpression promoted the nuclear accumulation, DNA binding activity, and transcriptional activity of NF-κB. Mechanically, PAQR3 directly interacted with IKKβ. The upregulation effect of PAQR3 overexpression on the expressions of FN and ICAM-1 was abolished by the treatment of NF-κB siRNA or PDTC (ammonium pyrrolidinedithiocarbamate) in HG-treated GMCs.

**Conclusion:**

PAQR3 promotes the expressions of FN and ICAM-1 *via* activating NF-κB signaling pathway. Mechanistically, PAQR3 activates NF-κB signaling pathway to mediate kidney inflammatory fibrosis through direct interaction with IKKβ in DN.

## Introduction

Diabetic nephropathy (DN), also known as glomerulosclerosis, is believed to be a common chronic microvascular complication of diabetes, and the most prevalent cause of middle-late renal fibrosis (1–3). The main pathological characteristic of DN is glomerular sclerosis resulting from microvascular pathological changes induced by diabetes. It is a leading cause of morbidity and mortality in patients with DN (4). Glomerular mesangial cells (GMCs), the intrinsic cells in glomeruli, play crucial roles in renal physiological functions and pathological changes (5–7). The accumulated extracellular matrix (ECM) components (such as fibronectin, FN) and inflammatory mediators (such as cell adhesion molecules, ICAM-1) in GMCs are involved in glomerular basement membrane thickening and glomerular fibrosis (3, 8, 9).

It is well documented that glycolipid metabolism disorders (10), non-enzymatic glycation of proteins (11), oxidative stress and cytokine secretion (12), polyol pathway (13, 14), and MAPK pathway (15) are all involved in the development and progression of diabetic renal fibrosis. Nevertheless, there are certain other mechanisms that may yet be investigated and defined. In recent years, lots of evidence highlighted the importance of inflammation in diabetic renal fibrosis, which attains our great concern (16–20). In diabetic kidneys, the activated NF-κB signaling pathway promotes the excessive expression of inflammatory mediators, which results in continuous or amplifying inflammatory responses and the secretion of ECM, eventually causing diabetic renal fibrosis. Therefore, it is of great importance to explore the mechanism of regulating inflammation in diabetic renal fibrosis.

Progestin and adipoQ receptor 3 is localized at the Golgi apparatus with seven-transmembrane helices. PAQR3 is also known as the Raf kinase trapping to Golgi, because it can function as a spatial regulator of Raf-1 kinase by sequestrating Raf-1 to the Golgi (21, 22). The expression of PAQR3 is different in various tissues of mice and humans, with higher level in skin, liver, kidney, and testicular tissue. Previous studies confirmed that PAQR3, a new tumor suppression gene, may regulate inflammation (23, 24). Numbers of evidence indicated that PAQR3-deficient mice are resistant to high-fat-diet (HFD)-induced obesity and hepatic steatosis, accompanied by improvement of insulin resistance and insulin signaling, which suggests that PAQR3 plays a vital role in the regulation of glycolipid metabolism (25–28).

Considering that DN is characterized by renal inflammatory fibrosis and PAQR3 regulates insulin resistance, glycolipid metabolism, and inflammatory response, we are highly concerned whether PAQR3 can regulate diabetic renal inflammatory fibrosis, eventually contributing to DN. Hereby, this study was aimed to investigate the effect and the underlying mechanism of PAQR3 on NF-κB signaling pathway and expressions of FN and ICAM-1 in HG-treated GMCs.

## Materials and Methods

### Reagents and Antibodies

D-Glucose was purchased from AMRESCO (Solon, OH, USA). Bovine serum albumin (BSA, Fraction V) was purchased from Mbchem (Shanghai, China). Penicillin and streptomycin were purchased from Life Technologies (Grand Island, New York, NY, USA). PDTC was purchased from Sigma–Aldrich Corporation (St. Louis, MO, USA). Antibodies against FN and ICAM-1 were purchased from Santa Cruz Biotechnology (Santa Cruz, CA, USA). Antibodies against NF-κB, PAQR3, and LaminB1 were purchased from Abcam (Cambridge, MA, USA). Antibody against α-tubulin was purchased from Sigma (St. Louis, MO, USA). Antibody against IKKβ was purchased from Cell Signaling Technology (Boston, MA, USA). Rabbit IgG was purchased from Beyotime (Haimen, China). Alexa Fluor 488 goat anti-rabbit IgG was purchased from Rockford (IL, USA).

### Cell Culture

Primary GMCs were isolated from glomeruli of Sprague–Dawley (SD) rats (about 150 g) and identified with specific assay (29). Briefly, the cortex fragments were cut into 1–2 mm pieces and sieved by specific mesh sizes (175, 147, and final 74 µm) mechanically. In the end, the filterable fragments were collected and digested with 0.1% collagenase IV in serum-free DMEM (Gibco, Carlsbad, CA, USA) for 20–30 min at 37°C. Then the digested matters were seeded in flasks with growth media (FBS, 20%; insulin, 0.66 U/mL; l-glutamine, 2 mM; 100 U/mL penicillin, and 100 U/mL streptomycin in DMEM), and incubated at 37°C under an atmosphere of 5% CO_2_. The GMCs were used at passages between the 5th and 12th and cultured in normal glucose DMEM (NG, 5.6 mM) with 10% FBS. GMCs were treated with serum-free DMEM for 12 h at 80% confluent and then treated with high glucose (HG, 30 mM) or other stimuli.

### Western Blot Assay

Western blot assay was performed with the standard protocol as previously described (29, 30) to detect the related proteins. GMCs or kidney tissues were harvested and lysed in RIPA lysis buffer [50 mM Tris pH 7.4, 150 mM NaCl, 1% NP-40, 0.5% sodium deoxycholate, 0.1% SDS] with protease inhibitor cocktail, phosphatase inhibitor A and B for 30 min. After centrifuged at 12,000 *g* for 15 min at 4°C, total proteins were collected. Besides the nuclear proteins and cytoplasmic proteins could harvest by a commercially available assay kit purchased from Active Motif (Carlsbad, CA, USA). Protein concentration was determined using a BCA™ Protein Assay Kit (Pierce, USA) following the protocol of the manufacturer. An equal amount of collective proteins from cells or tissues were separated by 8% sodium dodecyl sulfate-polyacrylamide gel electrophoresis. Then the proteins were transferred to PVDF membrane (Millipore, CA, USA) and blocked with 5% skim milk at room temperature for 1 h before being incubated with primary antibodies overnight at 4°C. After incubation, the membrane was washed with 0.1% Tween-20/TBS (TBST) and incubated with corresponding HRP-conjugated secondary antibodies (anti-rabbit IgG, anti-mouse IgG, or anti-goat IgG 1:10,000) at room temperature for 1 h. The signals were visualized with ImageQuant LAS 4000 mini obtained from GE healthcare (Waukesha, WI, USA) and then analyzed using the Quantity One Protein Analysis Software purchased from Bio-Rad Laboratories (Hercules, CA, USA).

### Immunofluorescence Staining

Glomerular mesangial cells were seeded on the glass cover slips. After transfection with plasmids or siRNA targeting PAQR3 for 24 h, the cells were washed with cold phosphate-buffered saline, fixed with 4% paraformaldehyde for 15 min at room temperature, permeabilized with 0.1% TritonX-100 for 10 min, blocked with 10% goat serum for 1 h, and then incubated with primary antibodies overnight at 4°C. After washing, the cells were incubated with fluorescent secondary antibody in the darkroom at room temperature for 1 h. The nuclei were labeled with 40, 6-diamidino-2-phenylindole (DAPI, Sigma, USA) for 10 min. Finally, the images were captured using a laser scanning confocal fluorescence microscope (LSM510, Carl Zeiss, Germany).

### Cell Transfection of Plasmids and Small-Interfering RNAs

Transfection of His-tagged PAQR3 plasmids was performed according to the manufacturer’s instruction using LTX reagent and PLUS™ reagent (Molecular Probes, Eugene, OR, USA). GMCs were cultured for 24 h prior to transfection, and then transfected with 2 µg of plasmids for 48 h. After further treatment, the cells were harvested for Western blot analysis.

Three pairs of small-interfering RNAs of PAQR3 or NF-κB were purchased from Gene Pharma (Shanghai, China). The most valid oligonucleotides of PAQR3 were si835 and their sequences were as follows: sense: 5′-GAUUGUGAUGUACGUGAUUTT-3′, antisense: 5′-AAUCACGUACAUCACAAUCTT-3′; the sequences of the most valid oligonucleotides of NF-κB were as follows: sense: 5′-GCUCGUGAGGGAUCUGCUATT-3′, antisense: 5′-UAGCAGAUCCCUCACGAGCTT-3′. GMCs were transfected with PAQR3 siRNA or NF-κB siRNA using RNAiMAX transfection reagent (Life Technologies, Grand Island, New York, NY, USA) according to the manufacturer’s protocol and then incubated for 48 h. After further treatment, the cells were harvested for Western blot analysis.

### Dual Luciferase Reporter Assay

Glomerular mesangial cells were seeded in 96-well plate and cotransfected with 0.2 µg pNF-κB-Luc (Beyotime, Haimen, China) and 0.02 µg pRL-TK (Promega, Madison, WI, USA) in the presence or absence of 0.05 µg of Penter-his-PAQR3. After treatment with HG, cells were harvested to analyze the luciferase activity by the Dual-Glo^®^ Luciferase Assay System kit (Promega, Madison, WI, USA). Luciferase activity was normalized to the renilla luciferase activity.

### Electrophoretic Mobility Shift Assay

Nuclear proteins were extracted by a nuclear extract kit, and the DNA binding activity of NF-κB was measured by EMSA (Thermo Fisher Scientific, Rockford, IL, USA) according to manufacturer’s instruction. The sequence of the biotin-labeled oligonucleotide probes for NF-κB was as follows: 5′-AGTTGAGGGGACTTTCCCAGG-3′. 6 µg of nuclear proteins were incubated with the mixtures containing 50 ng/mL poly (dIdC), 0.05% Nonidet P-40, 5 mM MgCl_2_, and 2.5% glycerol for 10 min. The mixtures were incubated with NF-κB probes for another 20 min at room temperature, separated by 6% non-denaturing PAGE, transferred to nylon membrane for DNA cross-links for 15 min, and then blocked for 1 h. After washed, the membrane was bound with horseradish peroxidase-conjugated streptavidin antibodies (1:300) for 15 min, last visualized and quantified with enhanced chemiluminescence by ImageQuant LAS 4000 mini (GE Healthcare, USA).

### Immunoprecipitation

After treatment with HG, GMCs were harvested and lysed with immunoprecipitation buffer on ice for 30 min. After centrifuged at 12,000 *g* for 10 min at 4°C, the supernatant was collected. 300 µg of proteins were incubated with 2 µg of test antibody or rabbit IgG overnight at 4°C with shaking. 20 µL of protein agarose A/G beads was added to that mixture for further incubation. After shaking for 2 h at 4°C, the beads were washed three times with immune-precipitation buffer 1, 2, and 3 successively. 15 µL of SDS loading buffer was added to that beads and boiled twice for 5 min. At last, immunoprecipitate was followed by Western blotting with respective antibodies.

### Animal Model

Animal experiments were carried out as previously described (30). Male C57/BL6 mice (*n* = 14) and SD rats (*n* = 14) were obtained from the Laboratory Animal Center, Sun Yat-sen University, Guangzhou, China. The experimental diabetic models (*n* = 7) were induced by intraperitoneal injection of freshly prepared STZ (40 mg/kg) in citrate buffer once a day for five continuous days. Control group (*n* = 7) were injected with an equal volume of citrate buffer (pH 4.5). Diabetic mice or rats with fasting blood glucose levels more than 11.1 or 16.7 mM were considered as experimental diabetic rodents, respectively. Diabetic rodents were fed with HFD for 8 weeks before their kidneys were collected for Western blot analysis. All experimental procedures were carried out in accordance with the China Animal Welfare Legislation, and approved by the Ethics Committee on the Care and Use of Laboratory Animals of Sun Yat-sen University.

### Statistical Analysis

All experiments were repeated at least three times. The data were assessed by the Graphpad Prism 5.0 software and values were expressed as mean ± SD. Data were analyzed by Unpaired Student’s *t*-test for comparison between two groups, and by one-way ANOVA with *post hoc* multiple comparisons for multiple comparisons. *P* < 0.05 was considered statistically significant.

## Results

### The Expression of PAQR3 Was Upregulated in Kidneys of Diabetic Animals or in HG-Induced GMCs

**Figure 1:**
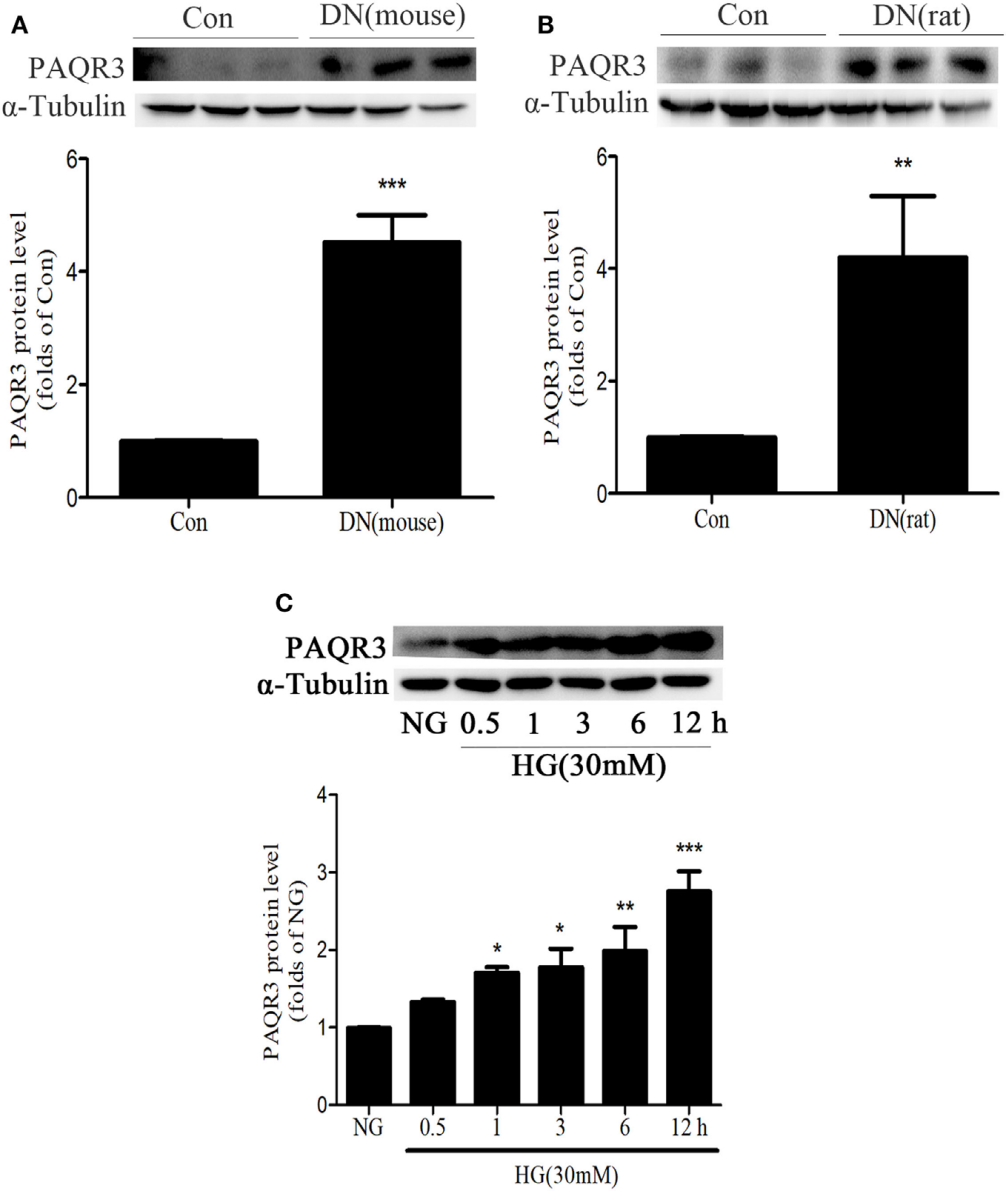
The expression of progestin and adipoQ receptor 3 (PAQR3) was upregulated in kidneys of diabetic animals or in high glucose (HG)-induced glomerular mesangial cells (GMCs). The expression of PAQR3 was assessed by Western blot in Con or the STZ-induced diabetic mice **(A)** and rats **(B)** kidneys. **P < 0.01, ***P < 0.001 vs. Con. After treatment of GMCs with 30 mM HG, the PAQR3 protein level was raised in a time-dependent manner (0, 0.5, 1, 3, 6, and 12 h) **(C)**. *P < 0.05, **P < 0.01, ***P < 0.001 vs. NG. Independent experiments were performed at least three times with similar results.

To determine the changes of PAQR3 expression in the kidneys of diabetic animals, we first examined PAQR3 protein level in STZ-induced diabetic kidneys by Western blot assay. Compared with the mice or rats in control group, PAQR3 expression was upregulated in diabetic rodents (Figures 1A,B) and up to 4.75- and 4.5-fold, respectively. Furthermore, HG stimulation enhanced PAQR3 protein expression in a time-dependent manner (Figure 1C). Therefore, these results indicated that the upregulation of PAQR3 may be involved in diabetic renal fibrosis.

### Overexpression of PAQR3 Induced the Expressions of FN, ICAM-1 in HG-Cultured GMCs

**Figure 2:**
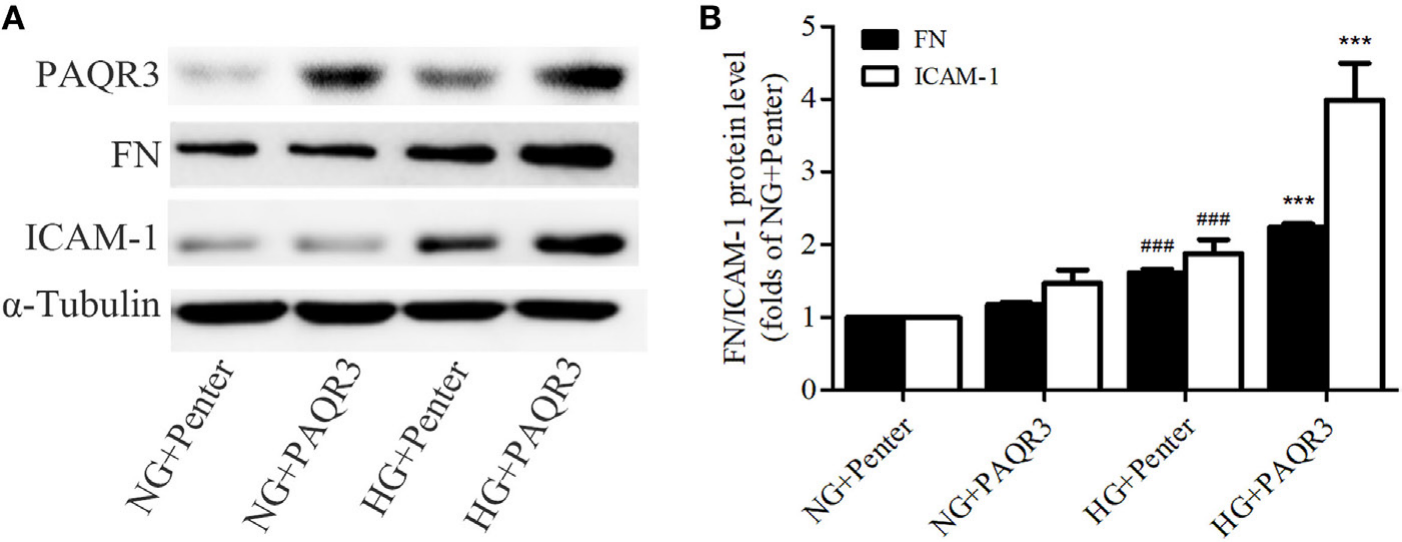
Overexpression of progestin and adipoQ receptor 3 (PAQR3) induced the expressions of fibronectin (FN), intercellular adhesion molecule-1 (ICAM-1) in high glucose (HG)-cultured glomerular mesangial cells (GMCs). GMCs were transfected with 2 µg of Penter vector or his-PAQR3, respectively. After treatment with HG for 24 h, total proteins were extracted to measure expressions of PAQR3, FN, and ICAM-1 **(A,B)**. ^###^*P* < 0.001 vs. NG + Penter, ***P < 0.001 vs. HG + Penter. Independent experiments were performed at least three times with similar results.

In the aforementioned experiments, we observed the upregulation of PAQR3 in diabetic kidneys and HG-treated GMCs. GMCs were transfected with plasmid expressing his-PAQR3. Our data showed that HG stimulation significantly increased the expressions of FN and ICAM-1, which was further increased by PAQR3 overexpression (Figures 2A,B). These results suggested that the upregulation of PAQR3 by HG promotes renal inflammatory fibrosis.

### PAQR3 Depletion Reduced the HG-Induced FN and ICAM-1 Expressions

**Figure 3:**
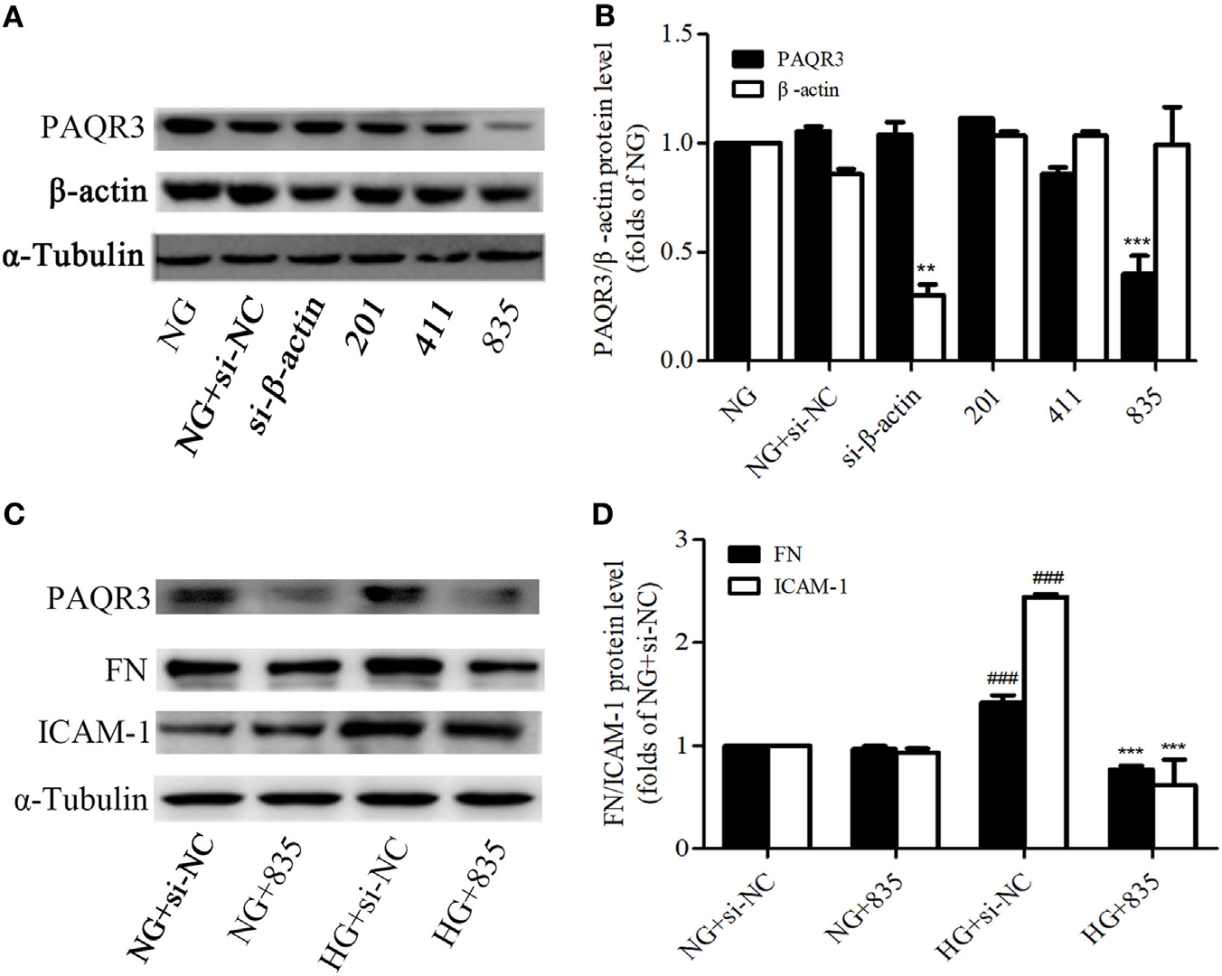
Progestin and adipoQ receptor 3 (PAQR3) depletion reduced the high glucose (HG)-induced fibronectin (FN) and intercellular adhesion molecule-1 (ICAM-1) expressions. Glomerular mesangial cells were transfected with negative control and three pairs of siRNA oligonucleotides targeting PAQR3 for 72 h, and then total proteins were harvested and subjected to Western blot assay. NC is a short form of negative control, and the depletion of β-actin protein acts as a positive control, which is used to evaluate the efficiency of PAQR3 depletion **(A,B)**. **P < 0.01 vs. NG, ***P < 0.001 vs. NG. PAQR3 depletion inhibited the upregulation of FN and ICAM-1 under HG conditions **(C,D)**. ^###^*P* < 0.001 vs. NG + si-NC, ***P < 0.001 vs. HG + si-NC. Independent experiments were performed at least three times with similar results.

Besides of studying the effect of exogenous PAQR3, we determined the effect of endogenous PAQR3 on FN and ICAM-1 expressions in GMCs under HG conditions as well. PAQR3 was knocked down by transfection of PAQR3 siRNA in GMCs. As results shown in Figures 3A,B, si835 could significantly reduce the PAQR3 protein level in GMCs. The upregulation of FN and ICAM-1 by HG was strikingly reversed after PAQR3 silencing (Figures 3C,D). These findings further suggested that PAQR3 may play an important role in DN by regulating FN and ICAM-1 expressions.

### HG Stimulation Enhanced NF-κB Nuclear Translocation in GMCs

**Figure 4:**
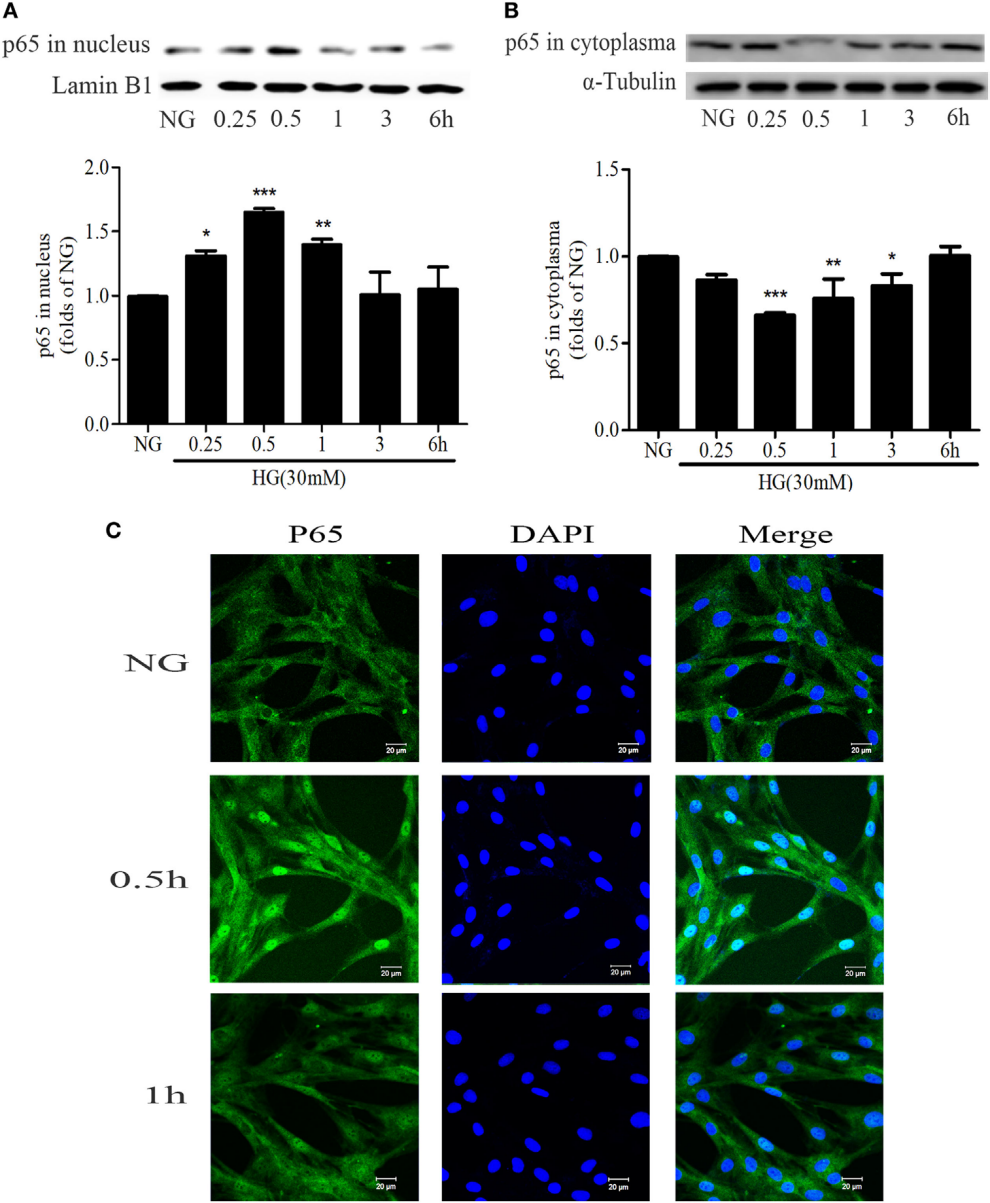
High glucose (HG) stimulation enhanced NF-κB nuclear translocation in glomerular mesangial cells. HG-induced nuclear accumulation of p65 reached a maximum level at 0.5 h, and then decreased at 1 h **(A,B)**. *P < 0.05, **P < 0.01, ***P < 0.001 vs. NG. Immunofluorescent staining showed the subcellular distribution of p65 in nuclei and cytoplasm at the indicated time points (0, 0.5, and 1 h) under HG conditions **(C)**. Blue and green stains indicate nuclei and p65, respectively. Bar: 20 µm. Independent experiments were performed at least three times with similar results.

NF-κB is an important nuclear transcription factor in mediating the inflammation as well as fibrosis lesions. Under HG conditions, NF-κB signaling pathway is activated in GMCs as demonstrated by increasing p65 nuclear translocation, promoting the transcription of downstream target genes. After treatment with 30 mM HG, the nuclear accumulation of p65 was increased. It reached a maximum level at 0.5 h and then decreased at 1 h (Figures 4A–C).

### Upregulation of PAQR3 Further Increased the NF-κB Nuclear Translocation, DNA Binding Activity, and Transcriptional Activity in GMCs

**Figure 5:**
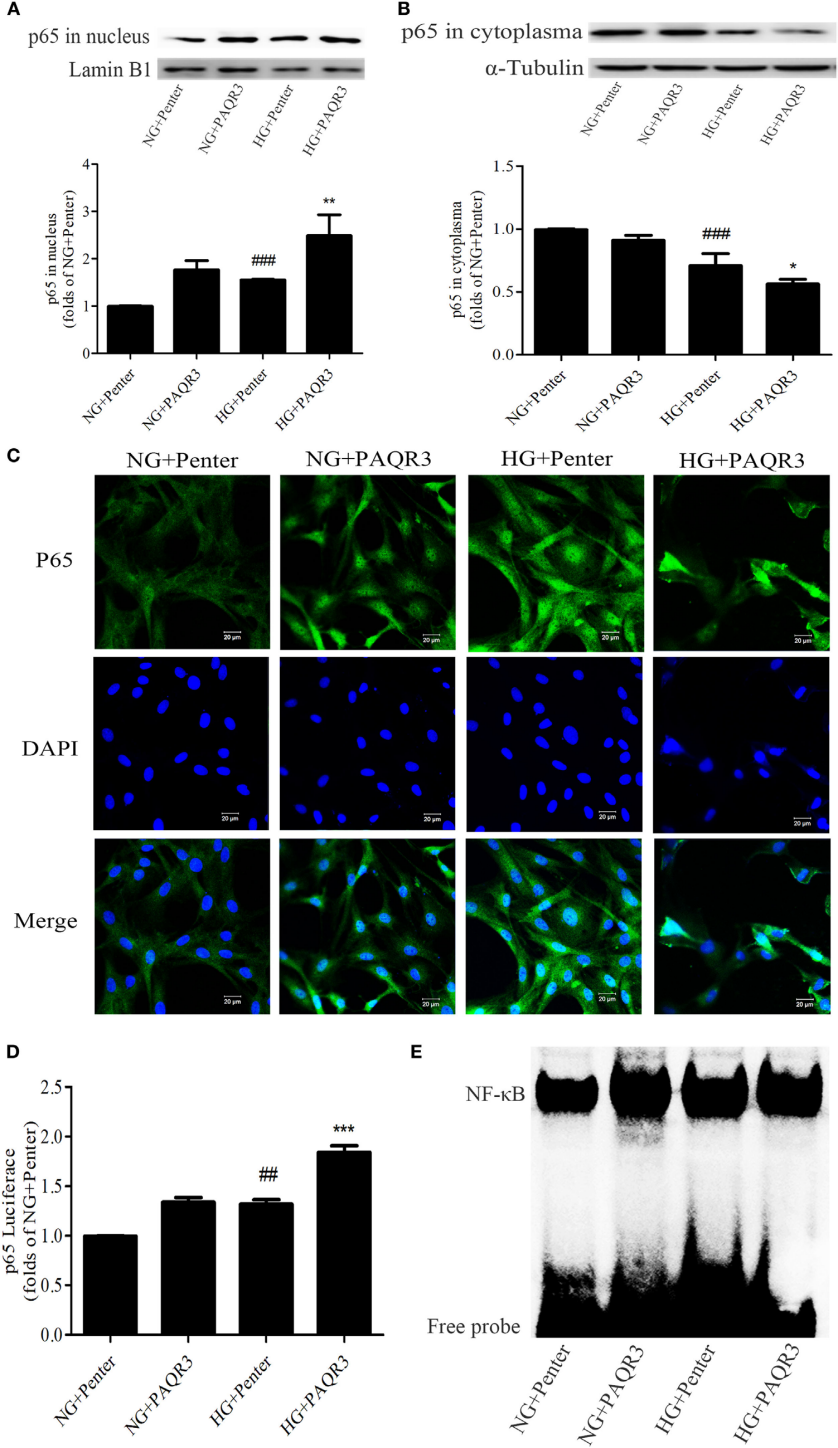
Upregulation of progestin and adipoQ receptor 3 (PAQR3) further increased the NF-κB nuclear translocation, DNA binding activity and transcriptional activity in glomerular mesangial cells. Overexpression of PAQR3 increased nuclear level of p65 **(A)** and decreased its cytoplasm level **(B)**. ^###^*P* < 0.001 vs. NG + Penter, *P < 0.05, **P < 0.01 vs. high glucose (HG) + Penter. PAQR3 overexpression enhanced the nuclear accumulation of p65, which was measured by immunofluorescent staining **(C)**. Bar: 20 µm. The effect of PAQR3 overexpression further enhanced the transcriptional activity of p65, which was obtained by luciferase reporter assay **(D)**. ^##^*P* < 0.01 vs. NG + Penter, ***P < 0.001 vs. HG + Penter. Electrophoretic mobility shift assay was performed to determine the elevated DNA binding activity of p65 **(E)**. Independent experiments were performed at least three times with similar results.

To explore the effect of PAQR3 on NF-κB signaling pathway in GMCs with HG stimulation, PAQR3 was overexpressed and NF-κB nuclear translocation, transcriptional activity, and DNA binding activity were detected. We found that the nuclear accumulation of NF-κB was increased under HG conditions, which was further augmented by PAQR3 overexpression (Figures 5A,B). This increased nuclear translocation was further confirmed in immunofluorescence images (Figure 5C). Dual luciferase reporter assay and EMSA also showed that transcriptional activity and DNA binding activity of NF-κB were further enhanced after overexpression of PAQR3 (Figures 5D,E). Taken together, our data indicated that overexpression of PAQR3 further activates NF-κB pathway in GMCs under HG conditions.

### PAQR3 Inhibition Suppressed the NF-κB Nuclear Translocation, DNA Binding, and Transcriptional Activity in GMCs

**Figure 6:**
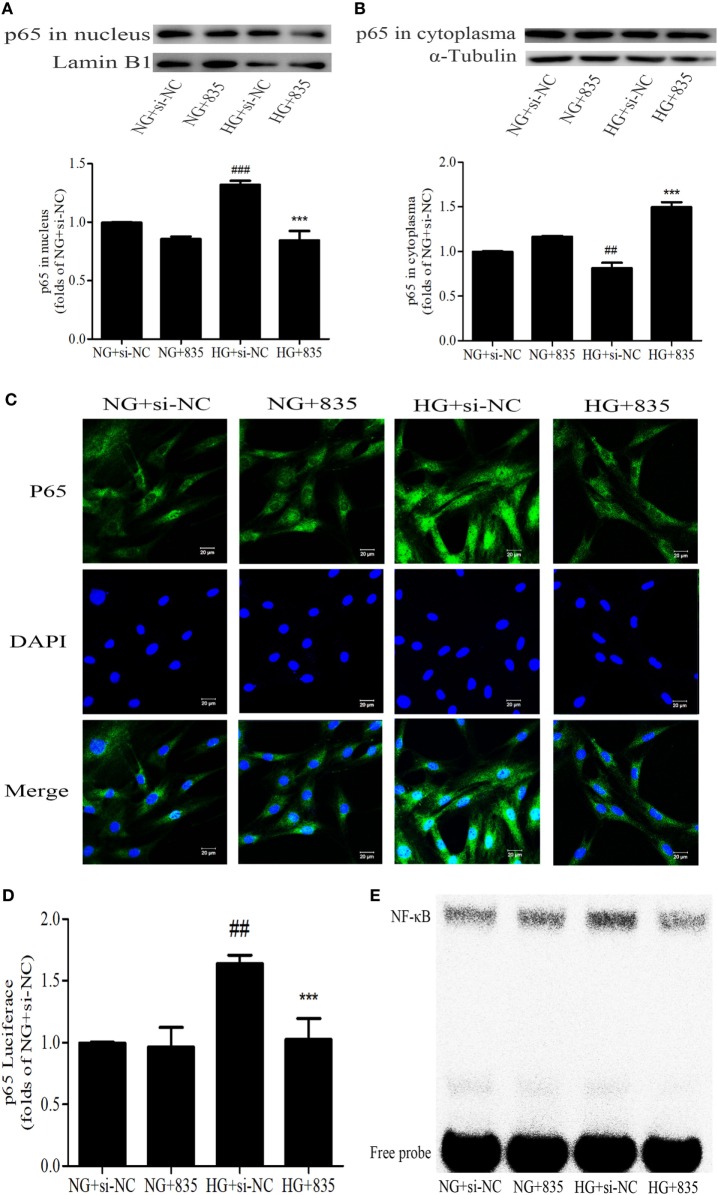
Progestin and adipoQ receptor 3 (PAQR3) inhibition suppressed the NF-κB nuclear translocation, DNA binding, and transcriptional activity in glomerular mesangial cells. High glucose (HG) stimulation increased p65 nuclear translocation, treatment with si835 decreased the nuclear level of p65 **(A)** and increased its cytoplasm level **(B)**. ^##^*P* < 0.01, ^###^*P* < 0.001 vs. NG + si-NC, ***P < 0.001 vs. HG + si-NC. HG-induced nuclear accumulation of p65 was prohibited under the conditions of PAQR3 inhibition **(C)**. Bar: 20 µm. HG stimulation for 12 h increased p65 transcriptional activity, which was inhibited by treatment with si835 **(D)**. ^##^ *P* < 0.01 vs. NG + si-NC, ***P < 0.001 vs. HG + si-NC. Knockdown of PAQR3 decreased the DNA binding activity of p65 compared with HG group **(E)**. Independent experiments were performed at least three times with similar results.

To further confirm the effect of PAQR3 on NF-κB pathway, the nuclear levels, DNA binding, and transcriptional activity of NF-κB were measured after silencing PAQR3. As expected, knockdown of PAQR3 suppressed HG-induced NF-κB nuclear translocation in GMCs (Figures 6A–C). Subsequently, the transcriptional activity (Figure 6D) and DNA binding activity (Figure 6E) of NF-κB were attenuated after PAQR3 knockdown, as confirmed by dual luciferase reporter assay and EMSA results. In general, our data indicated that PAQR3 positively regulates NF-κB signaling pathway in GMCs under HG conditions.

### HG Stimulation Attenuated the Interaction Between PAQR3 and IKKβ

**Figure 7:**
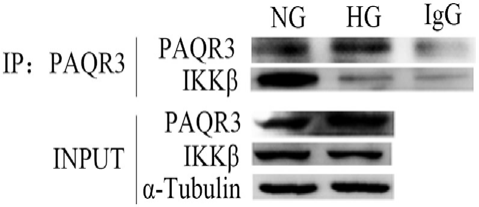
High glucose (HG) stimulation attenuated the interaction between progestin and adipoQ receptor 3 (PAQR3) and IKKβ. Immunoprecipitation assay was applied to detect the interaction between PAQR3 and IKKβ under NG and 1 h of HG treatment. Independent experiments were performed at least three times with similar results.

Progestin and adipoQ receptor 3 and NF-κB inflammatory signaling pathway all play important roles in the development of DN. To further study the relationship between PAQR3 and NF-κB, co-immunoprecipitation (Co-IP) assay was performed to investigate the mechanism underlying the regulation of PAQR3 on NF-κB signaling pathway. IKKβ is an upstream regulator of NF-κB signaling pathway, which can activate NF-κB pathway through phosphorylation of IκB (inhibitor of NF-κB) protein. By Co-IP assay, we found that PAQR3 interacted with IKKβ under physiological status. Moreover, this interaction was diminished after HG stimulation (Figure 7). Therefore, our data suggested that HG stimulation attenuates the interaction between PAQR3 and IKKβ, resulting in translocation of IKKβ to cytoplasm to phosphorylate IκB.

### Genetical and Pharmacological Inhibition of NF-κB Attenuated the Effects of PAQR3 Overexpression on HG-Induced Upregulation of FN and ICAM-1

**Figure 8:**
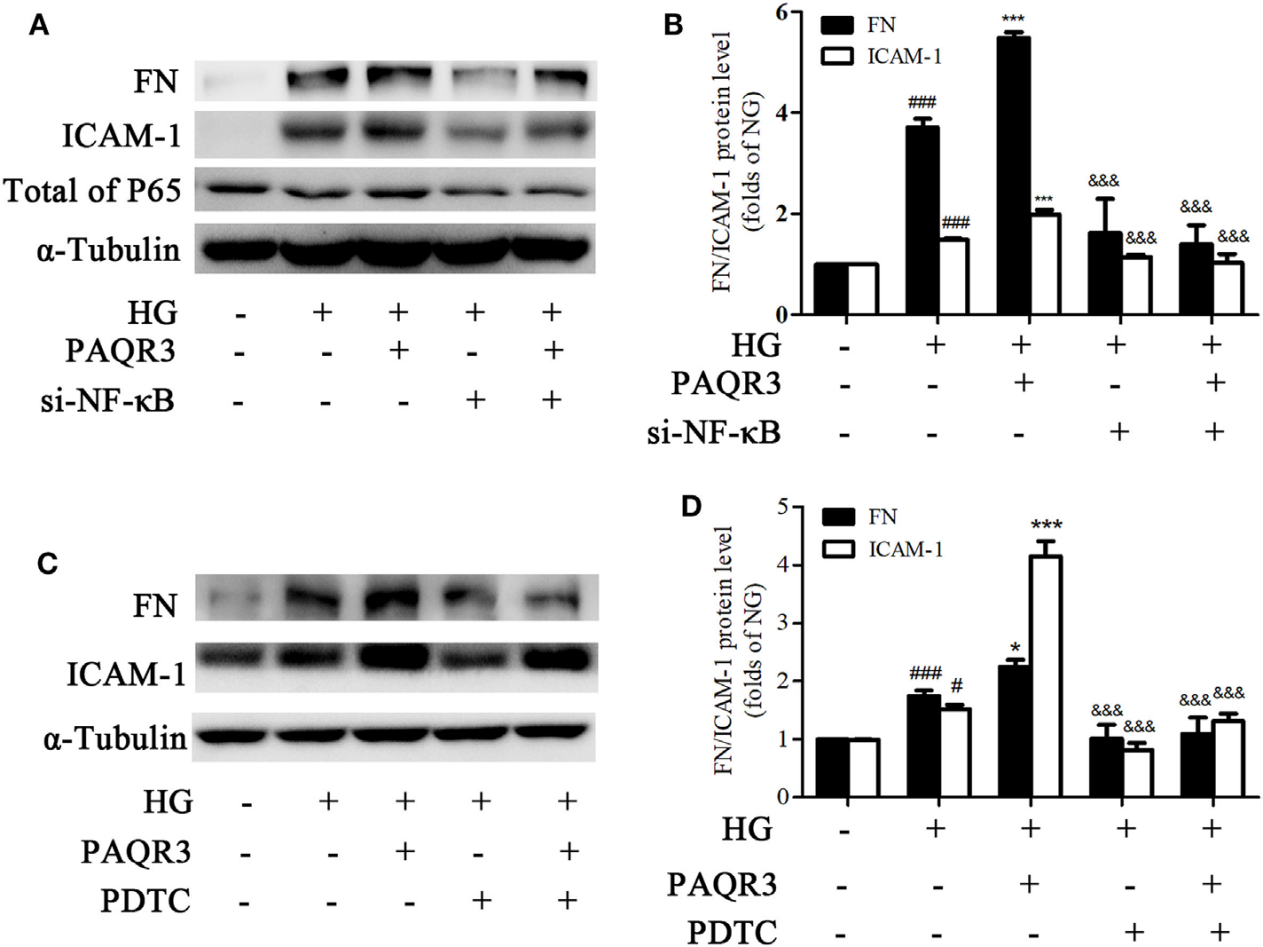
Genetical and pharmacological inhibition of NF-κB attenuated the effects of progestin and adipoQ receptor 3 (PAQR3) overexpression on high glucose (HG)-induced upregulation of fibronectin and intercellular adhesion molecule-1. Glomerular mesangial cells (GMCs) were transfected with si-NF-κB and PAQR3 plasmid for 24 h, then treated with HG for 24 h and total proteins were harvested to Western blot assay **(A,B)**. ^###^*P* < 0.001 vs. NG, ***P < 0.001 vs. HG, ^&&&^*P* < 0.001 vs. HG + PAQR3. GMCs were transfected with PAQR3 plasmid for 24 h, and then treated with PDTC (10 µM) under HG conditions for 24 h **(C,D)**. Total proteins were harvested to Western blot assay. ^#^*P* < 0.05, ^###^*P* < 0.001 vs. NG, *P < 0.05, ***P < 0.001 vs. HG, ^&&&^*P* < 0.001 vs. HG + PAQR3. Independent experiments were performed at least three times with similar results.

To further confirm whether NF-κB pathway was involved in the effect of PAQR3 on regulation of FN and ICAM-1, we both genetically knocked down NF-κB by using siRNA(Figures 8A,B), and pharmacologically inhibit NF-κB by using PDTC(Figures 8C,D), a well characterized inhibitor of NF-κB (31, 32). Overexpression of PAQR3 could further enhance the HG-induced upregulation of FN and ICAM-1 in GMCs. Transfection of si-NF-κB or treatment with PDTC alone significantly abrogated these effects. While GMCs were transfected with si-NF-κB or treated with PDTC in the presence of PAQR3 overexpression, the expressions of FN and ICAM-1 had no obvious changes compared with genetical and pharmacological inhibition of NF-κB alone. Therefore, our data suggested that PAQR3 mediates expressions of FN and ICAM-1 in DN *via* activation of NF-κB pathway.

## Discussion

PAQR family consists of 11 human PAQRs membrane proteins receptors, which can be grouped into three main classes, adiponectin receptors related subgroup (PAQR1-PAQR4), progesterone membrane protein receptors related subgroup (PAQR5-PAQR9), and the rest (PAQR10-PAQR11), based on the sequence comparisons (23, 33). PAQR3 is a type III topology receptor protein with a cytosolic N-terminus. Both N-terminus and loop structure are critical for PAQR3 to interact with signaling molecules in cytoplasm, thus to regulate the transmission of intracellular signals (22, 34). Since PAQR3 can be activated by adiponectin and shares high sequence homology with PAQR1 and PAQR2, it is classified into the adiponectin receptors related subgroup. PAQR3, as a new tumor suppressor gene, plays a vital role in inflammation, insulin resistance, glucose and lipid metabolic disorder diseases (23, 24, 35–39). It is also found that PAQR3 knockout obviously reversed HFD-induced diabetes, fatty liver, and remarkably improved insulin resistance, strengthen energy metabolism in mice (25). All of these evidences suggested PAQR3 plays an important role in the progression of diabetes and other diabetes-related complications.

Our study demonstrated that PAQR3 protein level was significantly increased by 4.75- and 4.5-fold in the kidneys of STZ-induced diabetic mice and rats, respectively. Also, HG stimulation increased the level of PAQR3 in GMCs in a time-dependent manner. While overexpression of PAQR3 further increased the HG-induced upregulation of FN and ICAM-1, PAQR3 silencing reversed these upregulation in GMCs. These results suggested that PAQR3 plays a role in the development of renal fibrosis in DN.

NF-κB signaling pathway is one of the classic inflammatory response pathways with the main function of regulating a large inflammatory gene in DN (20, 40). As we proved that PAQR3-silencing ameliorated the expressions of FN and ICAM-1, we wondered whether NF-κB pathway was involved in the regulation of PAQR3. As confirmed by multiple approaches, overexpression of PAQR3 increased nuclear accumulation, transcriptional activity, and DNA binding activity of NF-κB in HG-treated GMCs. In contrast, silencing of PAQR3 suppressed HG-induced augment of nuclear accumulation, transcriptional activity, and DNA binding activity of NF-κB in GMCs. Moreover, knockdown of NF-κB or treatment with NF-κB inhibitor, PDTC, abrogated the effect of PAQR3 overexpression on upregulation of FN and ICAM-1. In sum, our results indicated that PAQR3 regulates expressions of FN and ICAM-1 *via* NF-κB pathway.

However, the accurate mechanism how PAQR3 regulates activation of NF-κB pathway in DN remains to be elucidated. It was reported that IKKβ/NF-κB signaling pathway plays a major role in the metabolic inflammation (41, 42). IKKβ/NF-κB, as a mediator of metabolic inflammation, is a new strategy to combat obesity and its related diseases *via* regulating central insulin/leptin signaling and action (43–45). In light of reports that the IKKβ is required for the activation of NF-κB (46–48), we explored the interaction between PAQR3 and IKKβ in physiological status, or under the conditions of HG stimulation using co-IP. Here, we first reported the direct interaction between PAQR3 and IKKβ in physiological conditions, while the interaction was attenuated under conditions of HG stimulation. As it is well known that IKKβ is an upstream mediator of NF-κB signaling pathway, PAQR3 regulated NF-κB might *via* its interaction with IKKβ. However, the mechanism remained to be further explored. Considering that PAQR3 is also known as a Golgi-anchored membrane protein and Golgi apparatus is a workshop for proteins, we suspected that PAQR3 and IKKβ may co-exist in Golgi membrane as inactive forms. While stimulated by HG, IKKβ was separated from PAQR3 and moved to cytoplasm. Free and active IKKβ phosphorylated IκB to increase its degradation, resulting in translocation of NF-κB into nucleus to regulate the expression of downstream inflammatory genes.

In summary, our present study demonstrated that PAQR3 mediated pathogenesis of diabetic renal inflammatory fibrosis through NF-κB signaling pathway. Further investigation will be needed to determine if PAQR3 is a potential target for the treatment of diabetic kidney fibrosis.

## Ethics Statement

All experimental procedures were carried out in accordance with the China Animal Welfare Legislation, and approved by the Ethics Committee on the Care and Use of Laboratory Animals of Sun Yat-sen University.

## Author Contributions

YZ was mainly responsible for the idea of the article, conducting experiments, analyzing data, and writing articles. ZC participated in the language polish and article revision. JL participated in the data analysis and article revision. WG was mainly joined in experimental design and LZ involved in processing data mostly, and less other works. FX and LC major joined in experimental execution, and less other works. PL is our supervisor and involved in the idea of the article. HH is our supervisor. Besides, he was also involved in all parts of our research, such as providing ideas, instructing experiments and articles.

## Conflict of Interest Statement

The authors declare that the research was conducted in the absence of any commercial or financial relationships that could be construed as a potential conflict of interest.
